# Air pollution significantly associated with severe ocular allergic inflammatory diseases

**DOI:** 10.1038/s41598-019-54841-4

**Published:** 2019-12-03

**Authors:** Dai Miyazaki, Kazumi Fukagawa, Atsuki Fukushima, Hiroshi Fujishima, Eiichi Uchio, Nobuyuki Ebihara, Jun Shoji, Etsuko Takamura, Kenichi Namba, Yuichi Ohashi, Shigeki Okamoto, Yoshiyuki Satake, Hiroshi Ohtsu, Yumiko Shimizu, Yoshitsugu Inoue

**Affiliations:** 1 Division of Ophthalmology and Visual Science, Faculty of Medicine, Tottori University, Yonago, Japan; 2 Ryogoku Eye Clinic, Tokyo, Japan; 3 Kochi University, Kochi, Japan; 4 Tsurumi University School of Dental Medicine, Yokohama, Japan; 5 Fukuoka University, Fukuoka, Japan; 6 Juntendo University, Tokyo, Japan; 7 Nihon University, Tokyo, Japan; 8 Tokyo Women’s Medical University, Tokyo, Japan; 9 Hokkaido University, Hokkaido, Japan; 10 Ehime University, Toon, Japan; 11 Okamoto Eye Clinic, Matsuyama, Japan; 12 Tokyo Dental College, Tokyo, Japan; 13 Center Hospital of the National Center for Global Health and Medicine, Tokyo, Japan

**Keywords:** Conjunctival diseases, Risk factors

## Abstract

Ambient air pollution is a well-recognized risk for various diseases including asthma and heart diseases. However, it remains unclear whether air pollution may also be a risk of ocular allergic diseases. Using a web-based, nation-wide, cross-sectional study design, we examined whether the level of ambient air pollution is significantly associated with the prevalence of ocular allergic diseases. A web-based questionnaire was posted to invite the participants who are members of the Japan Ophthalmologist Association and their family members. The answers from 3004 respondents were used to determine whether there were significant associations between the level of the pollutants and the prevalence of ocular allergic diseases. The study period was between March to May 2017. The data of the air pollutants during 2012 to 2016 were obtained from the National Institute for Environmental Studies. The prevalence of allergic diseases was calculated by post stratification and examined for significant associations with the level of pollutants using multiple logistic regression analyses. The prevalence of seasonal allergic conjunctivitis, perennial allergic conjunctivitis, atopic keratoconjunctivitis (AKC), and vernal keratoconjunctivitis (VKC) in Japan was 45.4%, 14.0%, 5.3%, and 1.2%, respectively. The high prevalence of the severe forms of allergic conjunctivitis, including AKC and VKC, were significantly associated with the levels of the air pollutants. The prevalence of AKC was significantly associated with the levels of NO_2_ with an odds ratio (OR) of 1.23 (per quintile). The prevalence of VKC was significantly associated with the levels of NO_x_ and PM_10_ with ORs of 1.72 and 1.54 respectively. The significant associations between the prevalence of AKC and VKC and the levels of air pollutants indicate that clinicians need to be aware that air pollutants may pose serious risks of vision threatening severe ocular allergy.

## Introduction

Ambient air pollution is a well-recognized risk for various diseases including strokes, heart diseases, respiratory diseases, and lung cancer. For young children, air pollution is a significant threat to their general health. WHO estimates that approximately 1 in 10 deaths in children under the age of 5 years is caused by air pollution (https://www.who.int/news-room/detail/29-10-2018-more-than-90-of-the-world's-children-breathe-toxic-air-every-day) (2019). Numerous studies have found that air pollution aggravates asthma and can even trigger its onset^1^. However, very few studies have evaluated the relationship between air pollution and ocular allergic diseases.

Earlier studies have shown that ambient air pollution can aggravate conjunctivitis and allergic conjunctivitis^2^. During 1950–1970, photochemical pollution was prevalent in large cities such as Los Angeles and Tokyo^2^, and the pollution was suggested to be an urban environmental problem causing an allergic eye syndrome.

The air pollutants include particulate matter (PM), diesel exhaust particles (DEPs), and gases including NO, SO_2_, oxidants, and CO. The PMs are classified by their diameter; the PM_10_ have diameters of 2–10 µm, and the PM_2.5_ have diameters of 0.5–<2 µm. The PM_10_ include mainly dust, pollen, and mold, and the PM_2.5_ include combustion particles, organic compounds, and metallic particles^1^. The DEPs are very fine PMs and their diameter is <0.1 µm^3^.

In large cities, the major source of the pollutants is traffic-related, and the traffic-related air pollutants (TRAPs) are associated with asthma, allergy, and impaired lung development^4,5^. The TRAPs are a mixture of combustion-derived PMs, DEPs, and gaseous emissions, e.g., nitrogen oxides, CO, oxidants, and organic aerosols.

Atopic keratoconjunctivitis (AKC) and vernal keratoconjunctivitis (VKC) are the most refractory forms of the allergic ocular surface diseases, and they can cause significant visual morbidity. AKC and VKC often develop when eczema and systemic allergic diseases progress or steroid therapy fails. Thus, the onset and worsening of AKC and VKC are associated with the presence of systemic allergy and eczema.

There is good evidence that the prevalence of eczema is increasing, and one important factor for this is the increase of air pollution. For example, subjects in urban areas are more likely to be affected by eczema than those in rural areas^6^. In a nationwide study in Taiwan, the prevalence of flexural eczema and atopic dermatitis, was found to be associated with the ambient nitrogen oxides and carbon monoxide^7^. In a French study, a lifetime prevalence of eczema was associated with the level of ambient PM_10_, nitrogen oxides, and CO^8^. Mechanistically, NO_2_ and oxidants can induce direct chemical modifications to convert to allergens or associated molecules which can then directly damage skin barriers or be sensitizers for other allergens.

We hypothesized that ocular allergic diseases were significantly associated with air pollution. To test this, we conducted a survey of members of the Japan Ophthalmologist Association and their family members to determine whether the prevalence of ocular allergic diseases was significantly associated with the levels of air pollution.

## Results

Three thousand and four subjects were enrolled. The response rate was 10.8%. The mean age was 33.3 ± 18.6 years, and there were 1498 (49.9%) male participants. We first estimated the prevalence of diseases in Japan using data of the enrolled subjects after a finite population correction. For this, the demographic data of the population, age distribution, and the number of registered ophthalmologists by prefecture were used for poststratification.

The results indicated a high prevalence of affected allergic conjunctivitis of 48.7% which was comparable to that of allergic rhinitis (36.5%). Seasonal allergic conjunctivitis caused by cedar and cypress pollen was 37.4%. Perennial allergic conjunctivitis was the second most prevalent disorder at 14.0%, and that for AKC was 5.3% and VKC was 1.2%. When the systemic allergic diseases were assessed, the prevalence of atopic dermatitis was 7.0% and asthma was 5.8%.

**Figure 1:**
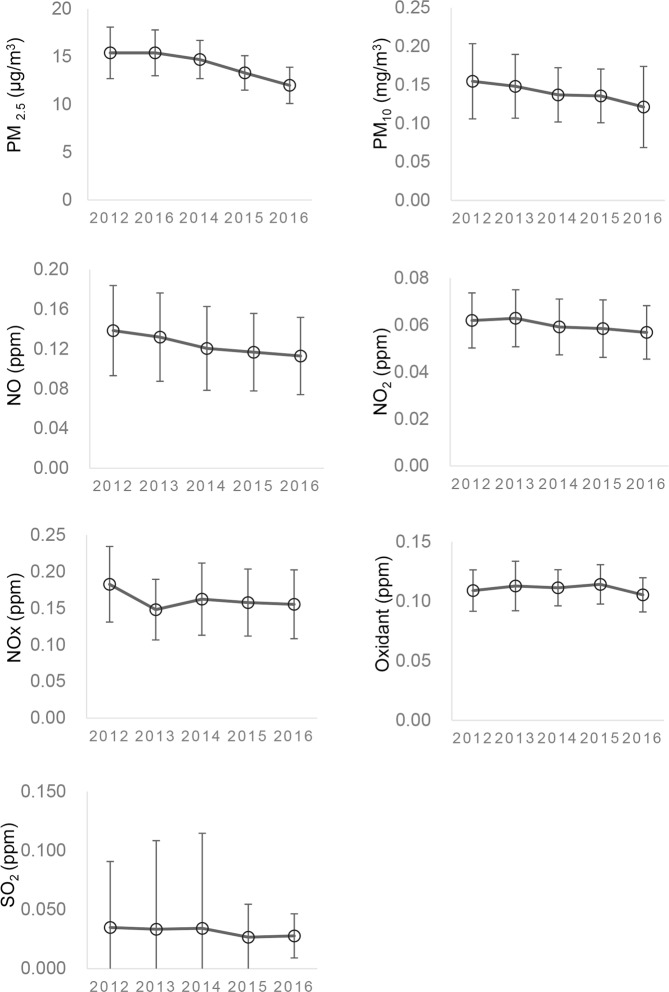
Levels of ambient air pollutants in 2012 through 2016. The average annual concentration of nitrogen oxides, NO, NO_2_, and NO_x_, and oxidant was within 0.1 to 0.2 ppm. Variation trends were almost stationary within recent years. The means of the maximum one-hour concentration of the day were used for PM_10_, NO, NO_2_, oxidant, and SO_2_. The annual 98% values of the day average concentration were used for NO_x_. The annual mean concentration was available for PM_2.5_.

We hypothesized that the increase of the TRAPs was significantly associated with the prevalence of severe ocular allergy. The well-known markers of the TRAPs are nitrogen oxides, oxidants, particulate matters (PMs), and carbon monoxide. We first assessed their nationwide levels in the 2012 to 2016 years using national surveillance data of air pollution from the National Institute for Environmental Studies, Japan (Fig. 1). The national average concentration of nitrogen oxides, NO, NO_2_, and NO_x_, and oxidant ranged from 0.1 to 0.2 ppm. Representative air pollution, including nitrogen oxides and PM_10_, were almost within the range of recommended levels by WHO Air Quality Guideline. (https://www.who.int/news-room/fact-sheets/detail/ambient-(outdoor)-air-quality-and-health) (2019). The trend pattern of their variations was almost stationary during the latest 5 years.

We then examined whether the prevalence of ocular allergic diseases was significantly associated with the air pollutant levels in the 2016 surveillance using logistic regression analyses (Table 1). The prevalence of each disease category was used as the dependent variable to calculate the odds ratios (ORs). The ORs were calculated after adjustments for age, cedar pollen dispersion, allergic rhinitis, and atopic dermatitis (Table 1). The highest OR for the air pollutants was observed for VKC. The second highest OR for pollutants was for AKC. This overall tendency was observed during 2012 through 2016 (data not shown).

**Table 1:** Association of ocular allergic diseases with air pollutants in 2016 by multiple logistic regression analysis.

	Vernal keratoconjunctivitis		Atopic keratoconjunctivitis	
	Odds ratio	P value	Odds ratio	P value
NO (quintile)	1.72	P = 0.002	1.21	P = 0.032
NO_2_ (quintile)	1.88	P = 0.003	1.23	P = 0.028
NOx (quintile)	1.72	P = 0.002	1.22	P = 0.029
Oxidant (quintile)	1.62	P = 0.017	1.13	P = 0.181
SO_2_ (quintile)	1.35	P = 0.059	0.93	P = 0.387
PM_2.5_ (quintile)	1.37	P = 0.192	0.81	P = 0.013
PM_10_ (quintile)	1.54	P = 0.023	1.10	P = 0.170
	**Seasonal allergic conjunctivitis**		**Perennial allergic conjunctivitis**	
	**Odds ratio**	**P value**	**Odds ratio**	**P value**
NO (quintile)	1.06	P = 0.256	0.98	P = 0.717
NO_2_ (quintile)	1.05	P = 0.427	1.04	P = 0.463
NOx (quintile)	1.07	P = 0.191	0.98	P = 0.736
Oxidant (quintile)	1.15	P = 0.078	0.90	P = 0.064
SO_2_ (quintile)	0.88	P = 0.021	1.13	P = 0.057
PM_2.5_ (quintile)	0.92	P = 0.257	0.98	P = 0.729
PM_10_ (quintile)	1.04	P = 0.378	0.97	P = 0.526

Adjusted for age, cedar pollen, allergic rhinitis, and atopic dermatitis.

For seasonal allergic conjunctivitis, the effects of the pollutants were weaker for VKC and AKC (Table 1). For perennial allergic conjunctivitis, the effects of the pollutants were not significant.

The levels of NO, NO_2_, NO_x_, oxidants, and PM_10_ were significantly associated with the prevalence of VKC with high ORs of 1.72, 1.88, 1.72, 1.62, and 1.54, respectively. They are summarized as nitrogen oxides-related pollutant. In contrast, the OR for SO_2_ was not significant during this period.

The levels of NO, NO_2_, NO_x_, and PM_2.5_ were significantly associated with the prevalence of AKC with ORs of 1.21, 1.23, 1.22, and 0.81, respectively. Thus, AKC was positively and significantly associated with the levels of the nitrogen oxides-related pollutants. However, a negative and marginally significant association was observed for PM_2.5_. Thus, the levels of nitrogen oxides-related pollutants were significantly associated with the prevalence of AKC, but their effects were different from that for VKC.

These findings were obtained after adjustments for systemic diseases and confounding variables. However, it is known that ocular and systemic allergic diseases are associated with each other, and each pollutant may affect the onset and symptoms of each disease differentially. Therefore, we began calculating their relationships considering the significant correlations of the ocular and systemic allergic diseases.

**Figure 2:**
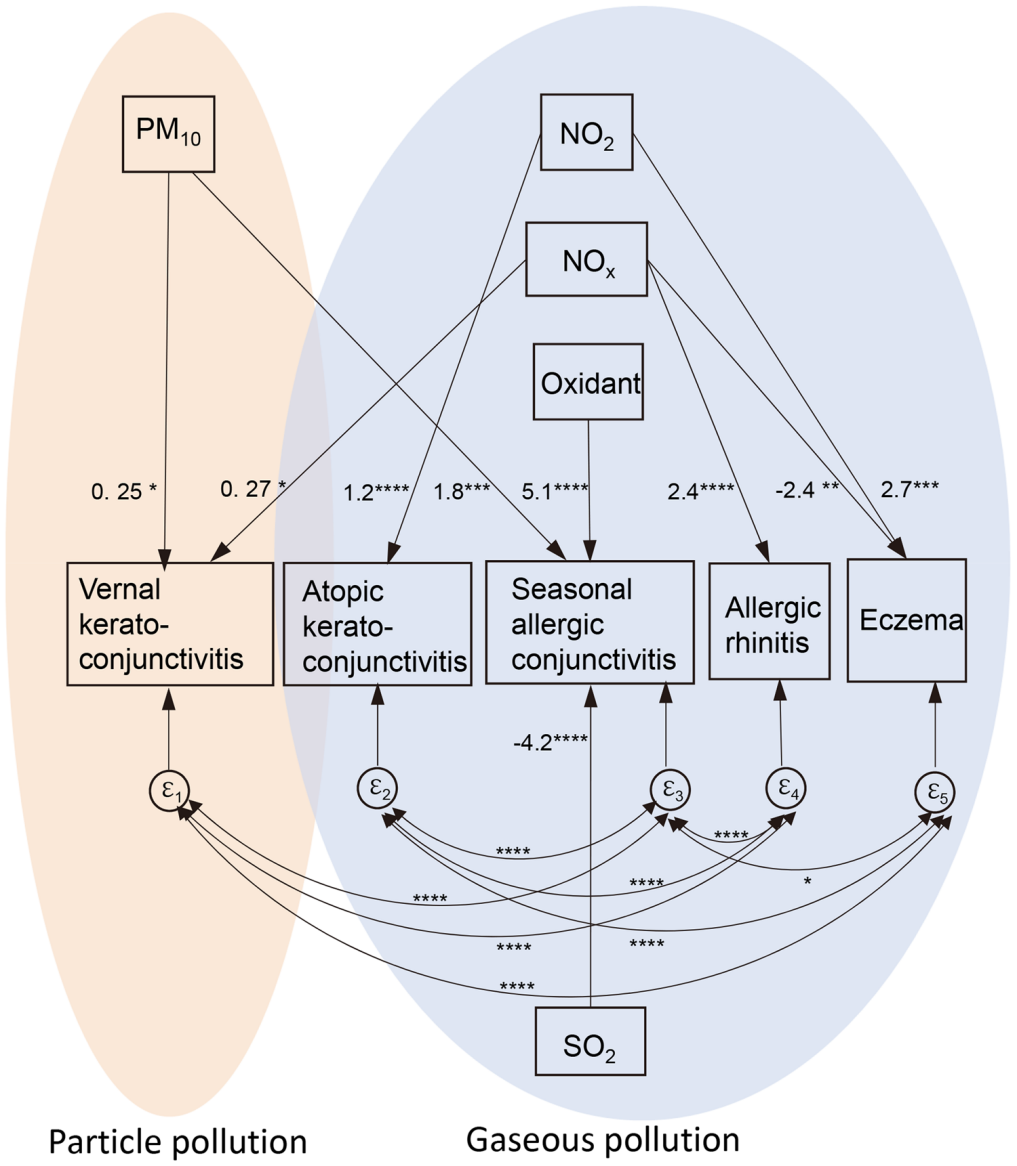
Association of ocular allergic diseases with particle or gaseous pollution by structural equation modeling analysis. The prevalence of ocular and allergic diseases was analyzed for associations with the annual levels of PM_10_, NO_2_, NO_x_, oxidant, and SO_2_ (per inter-quintile range) in 2016. The means of the maximum one-hour concentration of the day were used for PM_10_, NO_2_, oxidant, and SO_2_. The annual 98% values of the day average concentration were used for NO_x_. Values of arrows indicate significant co-efficient for disease prevalence expressed as percentage. Vernal keratoconjunctivitis was associated with particle pollutant, PM_10_ and gaseous NO_x_. Atopic keratoconjunctivitis was associated with NO_2_. Seasonal allergic conjunctivitis is positively associated with the levels of oxidant and PM_10_, but negatively associated with SO_2_. Allergic rhinitis was significantly associated with the level of NO_x_. Eczema was positively associated with NO_2_ but negatively with NO_x_. Significant covariance of error terms (ε) were observed between ocular and systemic diseases (double-headed arrow). The root mean squared error of approximation (RMSEA): 0.017, Comparative fit index (CFI) = 0.99. *P < 0.05, **P < 0.01, ***P < 0.005, ****P = 0.0000.

We first calculated the contribution of the air pollutants to the prevalence (%) of each allergic disease using multiple linear regression analysis. Based on the outcome of the multiple regression analysis (data not shown), we conducted a structural equation modeling (SEM) analysis with covariance structure (correlation) of the ocular and systemic allergic diseases (Fig. 2).

Consistent with the OR profiles calculated using multiple logistic regression analysis (Table 1), significant associations (arrows) were observed between the prevalence of the allergic diseases and the level of air pollutants. The air pollutants with similar effects were omitted from the analyses and are summarized for representative pollutants. The results indicated that VKC was significantly associated with particulate pollutant PM_10_ and gaseous NO_x_. In contrast, AKC and eczema were representatively associated with NO_2_. The numbers indicated are the coefficients of the regression equation. For example, a quintile increase of PM_10_ and NO_x_ adds 0.25 and 0.27 to 1.2 as the prevalence (%) of VKC. A quintile increase of NO_2_ adds 1.2 to 5.3 as the prevalence (%) of AKC.

Seasonal allergic conjunctivitis was positively associated with the levels of oxidant and PM_10_, and it was negatively associated with SO_2_ after adjustment of multiple air pollutants. The summed effect of the oxidants, PM_10_, and SO_2_ was small considering the very high prevalence of seasonal allergic conjunctivitis.

Significant correlations were observed for the error terms (ε) among the ocular and systemic allergic diseases. This indicated that what is unique about ocular and systemic diseases is correlated with what is unique about the others. Thus, their disease backgrounds are significantly correlated.

We also determined the association of cedar pollen dispersion with seasonal allergic conjunctivitis in the SEM analysis. The coefficient for pollen dispersion was −1.6% (per quintile, *P* = 0.003). Again, the relative contribution of cedar pollen seasonal allergic conjunctivitis was small and negligible. No other ocular allergic diseases had significant associations with the degree of pollen dispersion. When the effects on other ocular allergic diseases were calculated, the inclusion of pollen dispersion did not significantly affect the levels of the air pollutants coefficients (data not shown).

## Discussion

The results showed that air pollutants, especially the nitrogen oxides, were significantly associated with the more refractory forms of conjunctivitis, viz., VKC and AKC. Importantly, the nitrogen oxides were the representative air pollutants which have been shown to be associated with asthma in a number of cohort studies^9–11^.

Our nationwide survey showed that the prevalence of allergic conjunctivitis reached as high as 48.7% in Japan. Of these, the most prevalent form was seasonal allergic conjunctivitis which was mostly caused by cedar and cypress pollen in Japan. Previous reports indicated that the degree of aggravation of the symptoms was related to the numbers of air pollutants including NO, NO_2_, PM_2.5_, and PM_10_. In our study, seasonal allergic conjunctivitis had a significant association with the level of oxidants. The oxidants are generated from nitrogen oxides by photochemical reactions and can cause irritation as well as ocular surface damage.

Earlier studies showed that the levels of air pollutants were associated with an increase in out-patient visits for conjunctivitis. Of the pollutants, nitrogen oxide, ozone, and the PMs have been shown to be associated with an increase in the outpatient visits. A retrospective registry analysis in Shanghai, China showed elevated levels of NO_2_ and O_3_ and higher temperatures were associated with an increase in the outpatient visits for allergic conjunctivitis^12^. In a study conducted in Paris in 1999, the number of emergency visits for conjunctivitis and ocular surface problems was significantly associated with the ambient level of NO and NO_2_, high temperatures, and instant wind strength^13^.

These pollutants are strong irritants. Oxidant pollution is typically initiated by NO production mainly from automobiles. NO is then converted to NO_2_ followed by the generation of O_3_. All of these pollutants can directly damage the ocular surface by reducing the pH of the lacrimal fluid or by oxidization. Hot weather and strong winds, together with the dissemination of pollen allergens, can enhance these effects leading to instability of the ocular surface^13^. The increase in these pollutants may explain the increase of outpatient visits for allergic conjunctivitis. However, these pollutants are not essentially allergen specific although they will enhance such effects.

A significant association between the level of PMs and out-patient visits for conjunctivitis has also been reported. In a study of residents of Tokyo, PM_2.5_ was reported to be significantly associated with an increase of outpatient visits for allergic conjunctivitis^14^. A spatial analysis of residents of Daegu, Korea, showed that emergency visits for conjunctivitis and keratitis were significantly associated with the level of PM_10_^15^. In an analysis of pediatric patients in Lombardia, Italy, unspecific conjunctivitis was shown to be significantly associated with the level of PM_10_^16^. In a study in Taiwan (2007–2009), the number of outpatient visits for nonspecific conjunctivitis was significantly associated with increased levels of NO_2_, SO_2_, O_3_, and PM_10_^17^. Thus, the results of these studies indicated significant effects of PMs on the symptoms and signs of allergic conjunctivitis and conjunctivitis.

The larger PMs, such as PM_10_, consist of a number of possible allergens or adjuvants including pollen, bacteria, and fungi. This indicates that the PMs can provide antigen specificity to the response to the air pollutants. Thus, an increase of outpatient visits due to increases of PMs will reflect the recall responses of allergic symptoms as well as antigen non-specific augmentations.

In Japan, the prevalence of allergic conjunctivitis is very high and almost one-half of the population suffers from it. In our study, the prevalence of AKC was 5.3% and VKC was 1.2%. In the western European union, the prevalence of VKC was previously estimated as 0.03%^18^. However, a higher prevalence of VKC has been reported for Asian and African countries^19^. In southwest Ethiopia, the prevalence of VKC was estimated to be 5.2–7.3% in 2018, and it was significantly associated with dust exposure as a pollutant^19^. In our detailed analysis, AKC and VKC were significantly associated with the levels of ambient NO, NO_2_, NO_x_, and oxidants as well as PM_10_. NO_2_ was significantly associated with AKC with an OR of 1.23 (per quintile), and with VKC with an OR of 1.88 (per quintile).

As best we know, there have not been any studies that reported whether air pollutants, including the nitrate oxides, were significantly associated with the prevalence of allergic conjunctivitis. For the pollutants to be significantly associated with an increase of the prevalence, they need to act as allergen sensitizers. The results of previous studies indicated that nitrogen oxides and PMs may increase allergen sensitization^8–11^. In particular, the larger PMs can include a number of allergens although the nature of the allergen varies depending on the region of the country. In our analysis, a significant association was found between PM_10_ and VKC.

AKC and VKC generally occur in patients with atopic dermatitis or have an allergic disposition and also in those who have eczema, asthma, or rhinitis allergies. Thus, they may have already been sensitized. The correlations of disease backgrounds were confirmed in the SEM analysis (Fig. 2). Eczema and allergic rhinitis backgrounds were significantly correlated with the presence of VKC, AKC, and seasonal allergic conjunctivitis.

Based on the information gained from studies of asthma or eczema, there have been several mechanisms proposed for their association with air pollutants. One important mechanism was suggested to be through the activation of allergen sensitization by the air pollutants. NO_2_ is known to be associated with the prevalence of asthma and eczema^20^. Indeed, exposure to nitrogen oxides during early life was shown to significantly increase the risk of allergen sensitization in birth cohort studies conducted in Sweden, Netherland, and Canada^9–11^.

Other explanatory factors for allergic diseases included the effects of air pollutants on genetic or epigenetic effects. Oxidative stress is well known to provoke inflammatory responses leading to a worsening of allergic diseases. As for genetic factors, glutathione S-transferase P (*GSTP1*), which metabolizes reactive oxygen species, was reported to be significantly associated with air pollution and asthma^21^. NO_2_ associated risk of asthma was increased by the *GSTP1* variants, rs1138272 or rs1695. Thus, such populations are susceptible to asthma by air pollutants.

Epigenetic mechanisms are also known to affect the oxidative stress responses. A recent epigenome-wide association study showed that maternal NO_2_ exposure during pregnancy was significantly associated with DNA methylation of CpG sites in mitochondria-related genes of the newborn, which will then affect cellular stress responses^22^.

Besides the gaseous pollutants, the PMs are another important category of pollutants that are associated with allergic diseases. PM_2.5_ and PM_10_ are well known pollutants and have been reported to be associated with asthma and respiratory diseases^23–25^. In a French study of school children, PM_10_ was shown to be significantly associated with eczema, allergic rhinitis, and sensitization to pollen^8^.

We found that the prevalence of VKC was positively and significantly associated with the level of PM_10_. VKC is characterized by upper tarsal proliferative conjunctival and limbal lesions. These proliferative phenotypes may reflect an inflammatory response to the larger PMs which can include a number of allergens. This will be processed by their engulfment by macrophages for antigen presentation, leading to granulomatous or papillary lesions. In addition, PM_10_ may cause unregulated T cell responses and inflammation. For example, PM_10_ was shown to be significantly associated with reduced T_reg_ count in the EDEN birth cohort study^26^.

AKC was significantly associated with the level of nitrate oxides (Fig. 2). A direct toxicity of NO, NO_2_, NO_x_, or oxidants can cause inflammation or punctate keratitis which is characteristically observed in AKC. NO, NO_2_, NO_x_, and oxidants are mainly released as TRAP from road traffic which also contain DEP. The DEP can enhance NO_2_ or oxidant-induced allergic Th2 inflammation as a comorbid factor^27,28^.

There are some differences in the criteria used to diagnosis AKC and VKC from that proposed in European countries^29–31^. Generally, the diagnosis of AKC requires atopic dermatitis, however VKC occurs in young subjects with or without atopic dermatitis^30,31^. In the Japanese population, VKC is often observed in adults because VKC may be prolonged after puberty as a chronic condition. In European countries, AKC is generally considered to occur in adults. In the Japanese population, AKC with typical clinical characteristics is observed also from childhood. In addition, the incidence of both appears much higher in the Japanese or Asian population, and may be influenced by systemic allergy. In addition, their preposition will differ depending on the ethnicity and environment. Thus, the clinical characteristics and the background of the diseases appears to differ. Considering this, an exact comparison of their incidences needs be interpreted cautiously in Asian and European populations.

To adjust for the effects of systemic diseases, we conducted a SEM analysis with correlating error terms of ocular or systemic allergic diseases. These adjustments reduced the effects of interactions with systemic diseases which will be different depending on the ethnicity and environment.

There are some limitations in our study. We used the nationwide air pollutions statistics that were obtained from 1666 areas throughout Japan. We found that the level of air pollutants to be significantly associated with the prevalence of severe ocular allergic diseases after adjustments of possible confounding factors. However, our analysis was a cross sectional design which could not show causal relationships and may be affected by unobserved pollutants or variables.

To assess the actual risk of individual air pollutants to severe ocular allergy, a prospective design of birth cohort study is required. However, this requires much larger resources. In addition, our disease definition was based on the responding ophthalmologists of the family, and the response rate was 10.8%. This may have caused selection bias. However, estimated prevalence of allergic rhinitis and asthma by our study was 36.5% and 5.8%. These were reasonably consistent with reported prevalence of allergic rhinitis (39.4%, 2008) and asthma (5.4%, 2006)^32^ (https://www.erca.go.jp/yobou/zensoku/basic/glossary/kw75.html).

Importantly, our data may form the basis to conduct environmental monitoring, and we plan prospective studies in the future.

## Material and Methods

### Design and study population

We conducted a web-based, nationwide, cross-sectional study of members of the Japan Ophthalmologist Association and their family members. The study protocol was approved by Institutional Review Board of the Japanese Society of Ocular Allergology, and they conformed to the tenets of the Declaration of Helsinki.

A web-based questionnaire was presented to 8500 ophthalmologists between March 2017 and May 2017. An enrollment invitation was announced through the Japanese Society of Ocular Allergology and the Japan Ophthalmologist Association. The request was responded by 916 ophthalmologists, and the total number of subjects including their family members was 3004. All of the participants completed the questionnaire (Table 2). An informed consent was obtained from all the participants.

**Table 2:** Web-based questionnaire.

A. Primary residence	Selection of 47 prefectures in Japan
B. Age	Years
C. Sex	Male / Female
D. Presence of allergic conjunctivitis	1. None
	2. Seasonal allergic conjunctivitis (Cedar/cypress pollen)
	3. Seasonal allergic conjunctivitis (non- cedar/cypress pollen)
	4. Perennial allergic conjunctivitis
	5. Unknown
E. Presence of severe allergic conjunctivitis	1. None
	2. Atopic keratoconjunctivitis without proliferative lesion
	3. Atopic keratoconjunctivitis with proliferative lesion
	4. Vernal keratoconjunctivitis
	5. Giant papillary conjunctivitis
F. Presence of other allergic diseases	1. None
	2. Allergic rhinitis
	3. Eczema
	4. Asthma
	5. Food allergy
	6. Dry eye
	7. Others
	8. Unknown
G. Daily use of contact lens	1. None
	2. Disposable contact lens
	3. Non disposable soft contact lens
	4. Asthma
	5. Others
	6. Unknown
H. Use of topical medications for allergic conjunctivitis	1. None
	2. Histamine H1 antagonist eye drop
	3. Chemical mediator release inhibitor eye drop
	4. Steroid eye drop
	5. Cyclosporin or tacrolimus eye drop
	6. NSAIDs eye drop
	7. Others
	8. Unknown

### Assessment of allergic ocular diseases and comorbid factors

The questionnaire was designed to determine the prevalence of allergic ocular diseases and related factors in Japan (Table 2). The collected information were the: residence address of the subjects, age, sex, presence of allergic conjunctivitis (seasonal or perennial), presence of severe ocular allergic diseases (AKC, VKC, or giant papillary conjunctivitis), presence of systemic allergic diseases and related disease (allergic rhinitis, eczema, asthma, food allergy, dry eye), contact lens use, and use of topical allergic conjunctivitis medications (H1 blocker, mast cell stabilizer, steroid, calcineurin inhibitors, NSAID).

Respondents representing their families are ophthalmologists belonging to Japan Ophthalmologist Association or Japanese Society of Ocular Allergology. They followed a diagnosis guideline presented to them. The diagnosis of ocular allergic diseases was based on the published Japanese guideline for allergic conjunctival diseases 2017^30^.

### Acquisition of nation-wide air pollution survey data

The monitoring data for the air pollutants including the levels of NO, NO_2_, NO_x_, oxidants, SO_2_, PM_2.5_, and PM_10_ were obtained from the National Institute for Environmental Studies (http://www.nies.go.jp/db/index.html) (2019) for 2012–2016. Monthly monitoring data were obtained from 1666 monitoring stations covering all 47 prefectures in Japan. The annual averages and maximum hourly values were used for the analyses.

The monitoring data for cedar pollen dispersion for 2012–2016 were obtained from the Ministry of the Environment, pollen observation system (http://kafun.taiki.go.jp/) (2019). The monthly pollen count (/m^3^) was obtained from 120 monitoring stations throughout Japan.

### Statistical analyses

The prevalence of allergic diseases was calculated using a poststratification method. For this, the data of the registered number of ophthalmologist, age distribution, and population numbers of the 47 prefectures were obtained from the registry of the Portal Site of the Official Statistics of Japan (www.e-stat.go.jp/) (2019), and the estimated survey weight was calculated. The prevalence was calculated using the survey weight and adjusted for finite population correction.

The association between the pollutant levels and allergic diseases was analyzed by multiple linear regression and logistic regression models after poststratification and adjusted for the confounding variables. The odd ratios (ORs) were calculated for an interquintile range increase in the concentrations of the air pollutants.

A structural equation modeling analysis was conducted using the significantly contributing variables by multiple linear regression analysis to determine the contribution of air pollutants assuming covariance structure of ocular and systemic allergic diseases. The goodness of fit was evaluated using the root mean squared error of approximation (RMSEA), comparative fit index (CFI), and the Akaike’s information criteria (AIC). All of the analyses were conducted with Stata 15 (StataCorp, Tx). A *P* < 0.05 was considered significant based on a two-sided calculation. Values are expressed as the means ± standard deviations.

## Data Availability

The datasets generated during and/or analyzed during the current study are available from the corresponding author on reasonable request.
